# Behavioral and Neural Correlates of Acute and Scheduled Hunger in C57BL/6 Mice

**DOI:** 10.1371/journal.pone.0095990

**Published:** 2014-05-07

**Authors:** Christian M. Gallardo, Cynthia T. Hsu, Keith M. Gunapala, Maksim Parfyonov, Chris H. Chang, Ralph E. Mistlberger, Andrew D. Steele

**Affiliations:** 1 Division of Biology, California Institute of Technology, Pasadena, California, United States of America; 2 Department of Psychology, Simon Fraser University, Burnaby, British Columbia, Canada; 3 W.M. Keck Science Department, Claremont McKenna College, Pitzer College, Scripps College, Claremont, California, United States of America; 4 Biological Sciences Department, California State Polytechnic University Pomona, Pomona, California, United States of America; Pennsylvania State University, United States of America

## Abstract

In rodents, daily feeding schedules induce food anticipatory activity (FAA) rhythms with formal properties suggesting mediation by food-entrained circadian oscillators (FEOs). The search for the neuronal substrate of FEOs responsible for FAA is an active area of research, but studies spanning several decades have yet to identify unequivocally a brain region required for FAA. Variability of results across studies leads to questions about underlying biology versus methodology. Here we describe in C57BL/6 male mice the effects of varying the ‘dose’ of caloric restriction (0%, 60%, 80%, 110%) on the expression of FAA as measured by a video-based analysis system, and on the induction of c-Fos in brain regions that have been implicated in FAA. We determined that more severe caloric restriction (60%) leads to a faster onset of FAA with increased magnitude. Using the 60% caloric restriction, we found little evidence for unique signatures of neuronal activation in the brains of mice anticipating a daily mealtime compared to mice that were fasted acutely or fed ad-libitum–even in regions such as the dorsomedial and ventrolateral hypothalamus, nucleus accumbens, and cerebellum that have previously been implicated in FAA. These results underscore the importance of feeding schedule parameters in determining quantitative features of FAA in mice, and demonstrate dissociations between behavioral FAA and neural activity in brain areas thought to harbor FEOs or participate in their entrainment or output.

## Introduction

Circadian regulation of organismal physiology and health is a rapidly expanding area of research [1], [2], [3], [4]. Numerous studies have examined the entraining properties of light on the “master” clock, the suprachiasmatic nucleus (SCN), which is a cluster of ∼10,000 neurons in the most ventral and medial portion of the rostral hypothalamus with connections to many nearby nuclei involved in coordinating metabolism, wakefulness, and body temperature [5], [6], [7], [8]. Clearly, light inputs via the SCN can coordinate feeding behavior but the question of whether feeding behavior itself can be an important zeitgeber (entraining stimulus) has received increasing attention in recent years [1], [9].

If nocturnal rodents are fed during the day, the rest phase of their circadian rest-activity cycle, they show activity preceding the scheduled feeding time, termed “food anticipatory activity” (FAA), in addition to their normal nighttime activity [10]. In SCN-lesioned animals, which are arrhythmic when food is available *ad libitum* (AL), activity converges to a single peak preceding scheduled feeding, suggesting the presence of a timing mechanism that coordinates activity with feeding independent of the SCN [11], [12]. Formal properties of FAA in SCN-ablated and intact rodents are consistent with mediation by one or more food-entrainable circadian oscillators (FEO). Despite many years of investigation, the location of the FEO(s) necessary for FAA remains uncertain and food anticipatory rhythms are surprisingly robust to knockouts of known circadian clock genes [13], [14], [15], [16], [17]. The role of hunger related inputs assessed using gene knockout studies is no less murky [18], [19], [20], [21], [22], [23], [24].

A number of *ex vivo* neuronal activation studies have been undertaken to delineate the neuronal circuitry of FAA. Measures have included mRNA or protein expression of immediate early genes, such as *c-Fos*, or clock components like *Period 1 or 2*, or metabolic markers such as 2-deoxyglucose uptake in brain sections of rats or mice on daily feeding schedules [25], [26], [27], [28], [29], [30], [31], [32], [33]. These studies have broadly implicated several hypothalamic and a few extrahypothalamic regions as involved in FAA, with some variability in results across studies.

One potential cause of variable results is variation in the method and extent of food restriction. Previous studies mapping neural correlates of FAA have used limited-duration feeding schedules, in which rats or mice are permitted to eat as much as they can during a fixed mealtime, usually in the 2–6 h range. Here we present the results from experiments that utilized limited-amount feeding schedules, in which mice were provided once daily with an amount of food corresponding to some percentage of daily AL caloric intake. Mice on limited amount caloric restriction (CR) feeding schedules can eat for a more extended period of time, but still exhibit robust FAA. The first objective of this study was to determine the extent to which daytime FAA and nocturnal activity (which together determine the magnitude of commonly used metrics of FAA) depend on the degree of CR. We found that the rapidity and magnitude of FAA induction increases with the degree of CR, and established a 60% CR feeding schedule as optimal for inducing high amplitude FAA that is stable over many weeks. We then used a 60% CR schedule and immunohistochemical staining of the immediate early gene product c-Fos to map the neural correlates of CR at 5 time points before and after scheduled food access or an acute fast. We examined c-Fos expression in a number of brain regions implicated in FAA, including the nucleus accumbens, dorsomedial, ventromedial and lateral hypothalamus, and the cerebellum. Surprisingly, these areas did not exhibit significantly elevated c-Fos expression 1 and 2.5 h prior to scheduled daily food access, by comparison with acute fasting and AL food access conditions, although expression was marked 2 h after meal onset. These results indicate that limited-amount CR schedules can dissociate behavioral FAA from a commonly used correlate of neural activity in brain regions observed to be activated prior to limited-time feeding schedules.

## Materials and Methods

### Ethics Statement

These experiments were approved by the California Institute of Technology Institutional Animal Care and Use Committee under protocol #1567.

### Behavioral Analysis

Video-based activity data was analyzed using HomeCageScan 3.0; behavioral definitions were as described previously [34], [35]. High intensity activity was defined as walking, jumping, rearing, and hanging behaviors. Activity data were accumulated in 60-minute time bins and evaluated for statistically significant changes using non-parametric tests such as the Mann-Whitney Test using GraphPad Instat. All graphs were made with GraphPad Prism 4; medians are reported +/− interquartile ranges. Sample sizes are indicated in the Figure legends.

### Acute Fasting Experiment

For acute fasting experiments male C57BL/6J at least 10 weeks of age were purchased from Jackson Labs (Bar Harbor, ME). These mice were maintained on a 13∶11 LD cycle and single-housed for 4–6 days with AL access to food (Laboratory Rodent Chow Type 5001) and water prior to being placed on special feeding protocols. Daily food intake was measured over a 48 h period beginning at least three days after single housing and used to compute the amount of food delivered during CR (see below). For the experiment n = 13 mice were fasted and n = 15 mice were AL control. These mice were video recorded for 24 hours and the videos were analyzed using HomeCageScan 3.0.

### Dose-response Experiment

Male C57BL/6J mice from Jackson Labs were singly housed four days after arrival. Body weight measurements were taken at days −10, −3, 3, 7, 14, 21, 28, 35, and 42, with Day 0 marking the start of special feeding conditions (approximately twelve days after single-housing).

Mice were given AL food and water until day 0 of the study. Approximately one week prior to day 0, food intake was measured for forty-two mice over the course of two days. Starting from day 0, 2 hours prior to lights-off (Zeitgeber Time 10, where ZT12 is lights-off by convention) eight mice were given AL access to food, eight were given 110% of the mean food intake for all mice, eight were given 80% of the mean food intake for all mice, and ten were given 60% of the mean food intake for all mice. All mice were recorded for 23.5–24 hours once a week starting with day 0 and ending at day 42. Mice were fed and recorded at ZT 10. Water remained freely available throughout the study though we noted that mice did most of their drinking behavior during the 2 hours after meal presentation.

### Neural Correlates of Acute and Scheduled Hunger

Mice were divided into three groups: AL, CR, and Fast (no food for the indicated time before perfusions). Mice in AL and Fast groups had free access to chow and water. Mice in the CR group were provided with 60% of their AL levels. On the last day of the scheduled feeding (day 28), mice were euthanized with carbon dioxide and perfused transcardially with 10 mL of 0.1 M PBS at 5 time points relative to scheduled feeding: time point (TP) −7.5, −4.5, −2.5, −1, +2. Biological samples sizes for were n = 2 per group at TP −7.5; n = 4 for AL and CR and n = 2 for fasted at TP −4.5; n = 4 for AL and CR and n = 2 for fasted at TP −2.5; n = 3 for AL and CR and n = 2 for fasted at TP −1; and n = 2 for all groups at TP +2. The brains were removed from mice and immersion fixed in 10% buffered formalin (Sigma) for at least 24 h. They were then bisected in a saggital plane and half-brains were sent to NeuroScience Associates (Knoxville, TN). Samples were then treated with 20% glycerol and 2% dimethylsulfoxide to prevent freeze-artifacts and embedded with up to 32 hemi-brains in a block of gelatin matrix using MultiBrain Technology™. The block of embedded tissue was allowed to cure and then was rapidly frozen by immersion in isopentane chilled to –70°C with crushed dry ice. Blocks were mounted on a freezing stage of an AO 860 sliding microtome and sectioned coronally at 35 µm thickness. All sections cut were collected sequentially into a 4×6 array of containers filled with ‘antigen preserve’ (buffered ethylene glycol). At the completion of sectioning, each container holds a serial set of one-of-every-24th section (e.g. one section every 840 µm). For Immunostaining every 6^th^ section was used.

For immunochemistry, the sections were stained free-floating. All incubation solutions from the blocking serum use Tris buffered saline (TBS) with Triton X100 (TX) as the vehicle; all rinses are with TBS. After a hydrogen peroxide treatment and blocking serum, the sections were immunostained with a primary anti-c-Fos (Novus, rabbit anti c-Fos) 1∶10,000 antibody overnight at room temperature. Vehicle solution contains 0.3% TritonX-100 for permeabilization. To visualize the location of binding site of the primary antibody an avidin-biotin-HRP complex (details in Vectastain elite ABC kit, Vector, Burlingame, CA) is applied. After rinses, the sections were treated with diaminobenzidine tetrahydrochloride (DAB) using nickel enchancemnet and hydrogen peroxide to create a visible reaction product and mounted on gelatinized (subbed) glass slides, air dried, dehydrated in alcohols, cleared in xylene and cover slipped.

c-Fos stained sections were examined under a Nikon Eclipse TE2000-U light microscope coupled to a computer with NIS-Elements BR 3.0 software. Image saturation was avoided by adjusting exposure time with the brain slice with the highest c-Fos signal. Expression analysis of c-Fos protein was done for the following regions: SCN (0.48 to 0.655 caudal to bregma), nucleus accumbens core (NAc) (0.745 to 0.445 rostral to bregma), paraventricular hypothalamus (PVH) (0.48 to 0.655 caudal to bregma), Rostral arcuate (Arc) (1.055 to 1.855 caudal to bregma), caudal arcuate (Arc) (2.055 to 2.255 caudal to bregma), ventromedial hypothalamus (VMH) (1.255 to 1.755 caudal to bregma), dorsomedial hypothalamus (DMH) (1.255 to 1.755 caudal to bregma), lateral hypothalamus (LHA) (1.255 to 1.755 caudal to bregma), cerebellum (6.355 to 7.055 caudal to bregma), and superior colliculus (SuG) (3.68 to 4.45 caudal to bregma). Cells immunopositive for c-Fos protein were counted using 10x and 4x lenses. The mean number of c-Fos-positive cells per time point was calculated from individual unilateral counts at three contiguous bregma depths (with at least 3 replicates per time point). Counts were done automatically by software after setting an ROI and threshold levels for each brain region, with threshold levels kept constant in between counts of a specific brain region. ROIs were defined in slices based on visible landmarks and comparison with mouse brain atlas of Paxinos and Franklin (2007). Representative images were captured using the CCD camera while doing automated counts. Tukey-Kramer parametric regressions were used to establish statistical differences between groups (AL, CR, Fast).

## Results

### Acute Food Deprivation Increases Nocturnal Activity

**Figure 1:**
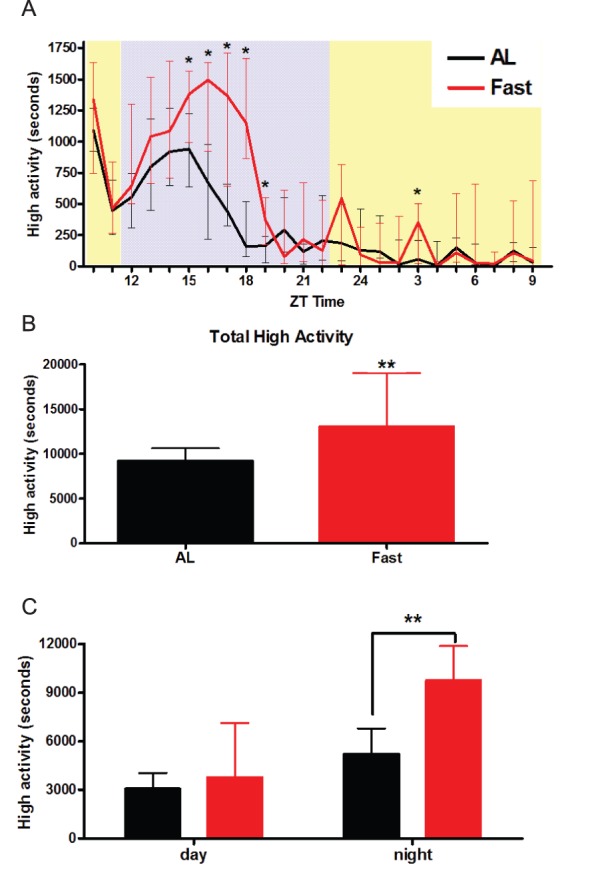
High activity profiles, and total diurnal and nocturnal activity of ad libitum and acutely fasted mice. (A) The seconds of high activity per hour in a 24 hour video recording of AL mice (black) or acutely fasted mice (red). The light phase is shown in yellow (ZT 10–12) followed by 11 hours of darkness indicated in gray (ZT 12–23) followed by light (ZT 24–9) (B) The total high activity summed across the 24 hour recording for AL and fasted mice. (C) The total high activity during the light and dark periods. n = 13 fasted and n = 15 AL. Values represent medians +/− IQ range. The statistical significance was tested using Mann-Whitney test; **denotes p<0.01.

We tested the home cage behavioral response to acute food deprivation in C57BL/6 male mice. At ZT10 (2 hours before lights-off) we removed all food from the cage of n  = 13 mice and left in ample food for n = 15 mice and monitored these mice for 24 hours. The fasted mice showed a strong increase in ‘high’ activity behaviors (defined as jumping, hanging, rearing, and walking), particularly in the middle-to late night hours of ZT 15–18 (Figure 1A). The total high activity over 24 hours was significantly increased in the fasted group compared to the AL control group during the 24-hour video recording (Figure 1B; p = 0.0044, Mann-Whitney). Activity during the 13 hour lights on period was not increased in fasted mice; however, nighttime activity almost doubled in fasted mice compared to AL controls, showing a statistically significant increase (Figure 1C; p<0.01, Mann-Whitney).

### Nocturnal Activity Decreases While FAA Increases with Increased Magnitude of CR

There is evidence that CR increases activity overall but it has not been examined in detail using C57BL/6J male mice under the same time scales typically employed by our laboratory [36], [37], [38]. To that end, we tested the effect of varying the percentage of food delivered on a timed CR schedule in C57BL/6J male mice and assayed their overall, nocturnal, and preprandial high activity behaviors.

**Figure 2:**
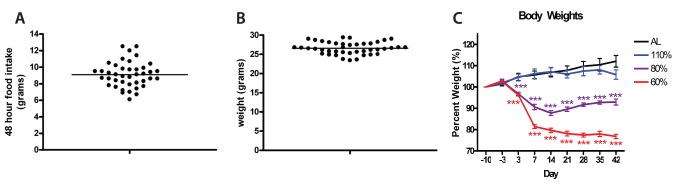
Food intake and body weight distribution and normalized body weights of mice on different food restriction amounts. (A) Individual measurements of food intake over a 48 hour period for individual mice divided by 2 to give a 24 hour food intake (n = 42). (B) Individual measurements of body weight prior to any dietary change (day−10) (n = 42). (C) Mean body weight measurements +/− SEM of each treatment group over the duration of the experiment. Mice fed AL (black; n = 8), 110% (blue, n = 8), 80% (purple; n = 8) and 60% (red; n = 10) show dose-dependent changes in body weight depending on food availability. Significance was tested with Tukey-Kramer multiple comparisons test with one-way ANOVA post-test for all treatment groups vs. AL controls for each respective day. ***denotes p<0.001.

We measured food intake over 48 hours for all mice used in this study, finding a broad range of values from 3 to 6 grams per day (mean 4.6+/−0.1 SEM; Figure 2A). Body weight showed some variability as well but not as much as food intake. The smallest mouse weighed 23.4 grams while the largest mouse weighed 29.3 grams (mean weight 26.6+/−0.2 grams; Figure 2B). Rather than scale the percent CR on an individual mouse basis, which would be cumbersome, we restricted based on the population average. For mice fed an AL diet we delivered an additional pellet to their cage daily at ZT 10 to control for the disturbance of feeding while for another group we delivered 110% of AL food intake (in pilot studies we observed FAA in some mice for 100% diets). Mice that were fed 60% of the mean food intake exhibited a precipitous decrease in weight until day 7, when the decrease tapered to ∼80% of day −3 baseline values (Figure 2C). Mice fed 60% CR also had significantly lower percent weight than those fed 80% CR starting on day 7 and lasting the duration of scheduled feeding experiments (p<0.001). Mice that were fed 80% CR had a significant decrease in weight from day −3 to day 14 (p<0.001); however, they also had a significant weight gain from day 14 to day 28 (p<0.001). Mice fed 110% of the mean food intake showed a modest increase in body weight that was similar to that of AL controls, until day 14, when body weight stabilized to around 105% of baseline value. Mice fed 110% did not show any statistically significant difference from mice fed AL in the percent change in weight relative to day −3. Mice fed AL had a significant change in weight between day −3 and day 14 (p<0.05), day 3 and 35 (p<0.05), day 3 and 42 (p<0.01), and day 7 and day 42 (p<0.01) (Repeated Measures ANOVA with Tukey-Kramer Multiple Comparison’s Test).

**Figure 3:**
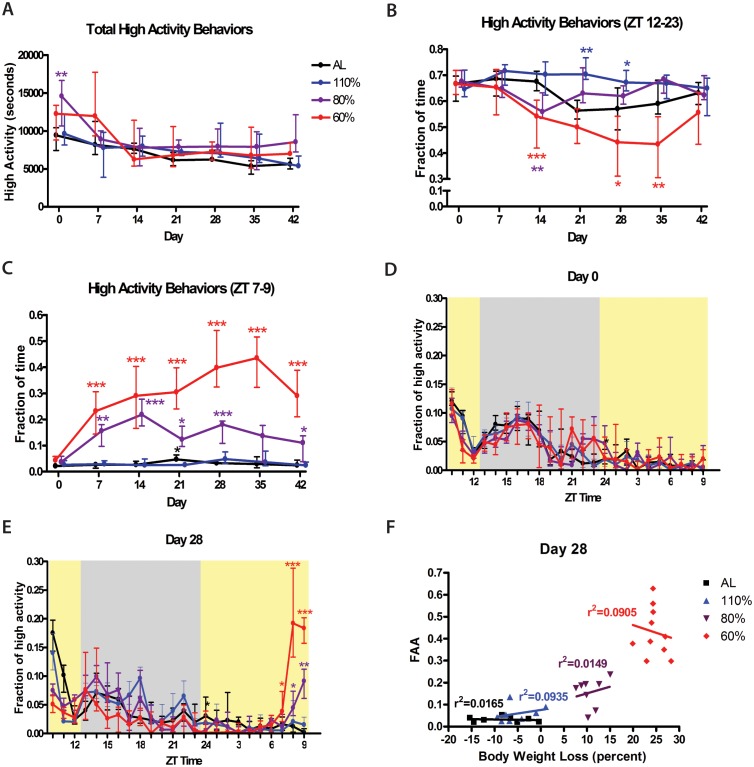
High activity behaviors of mice in on different food restriction amounts. (A) The total amount of high activity behaviors (in seconds) during weekly 24 hour video recordings for mice on AL, 110%, 80%, and 60% CR. (B) Normalized nocturnal high activity behaviors during weekly 24 hour video recordings for mice on AL, 110%, 80%, and 60% CR. (C) Fraction of time spent on high activity behaviors in the 3 hours preceding meal presentation (ZT 7–9). (D) Normalized fraction of time per hour spent on high activity behavior during the 24-recording of day 0 (D) and (E) day 28 of dose-response experiment. Values represent medians +/− IQ range, with high activity values normalized to total daily activity (significance tested with Mann-Whitney test with Dunn’s post test for all treatment groups vs. AL controls for each respective day). *denotes p<0.05, **denotes p<0.01, and ***denotes p<0.001. n = 8 for all groups except for 60% CR where n = 10. (F) Correlation and linear regression of normalized food anticipatory activity in the 3 hours preceding scheduled feeding with % body weight loss.

Overall, the total amount of high activity behaviors (in seconds) of mice on CR was not higher than that of AL controls (Figure 3A). In fact, we were surprised to observe that activity was significantly elevated only at one time point for 80% CR–the first day of the modified diet (“day 0”)–and that at no other points were mice on CR more active than those on AL or 110% AL control diets (Figure 3A). Despite this lack of a major difference in overall activity, we followed our standard practice of normalizing high activity by dividing the seconds of high activity in each hourly bin by the total high activity for that day, resulting in a fraction of high activity per hour. When binning for high activity behaviors during the 11 hours of darkness (ZT 12–23), mice in the 60% CR group showed a clear decrease in normalized nocturnal high activity, reaching its lowest points on days 28 and 35 (Figure 3B). Even when taking into account the slight decrease in dark cycle activity in AL controls over time, the decrease in nighttime activity in the 60% CR groups was statistically significant on days 14, 28, and 35 (p<0.001, p<0.05, and p<0.01 respectively). Conversely mice fed 110% of the baseline food intake showed a distinctly more constant fraction of nighttime activity compared to AL controls with statistically significant differences on days 21 and 28 (p<0.01 and p<0.5 respectively). Mice in the 80% CR group displayed a transient decrease in nighttime activity relative to the AL controls at day 14 but then showed a similar amount of nocturnal activity at all later time points (Figure 3B).

Preprandial high activity behaviors for CR groups during the 3 hours preceding scheduled meal time (ZT 7–9) showed a dose-dependent increase in the fraction of high activity compared to AL controls, with mice on 60% CR exhibiting an overall greater magnitude of FAA compared to those on 80% CR (Figure 3C). Mice on 60% CR showed FAA that was statistically significant (p<0.001) on all days following the start of restricted feeding schedules (Figure 3C). Mice on 80% CR displayed FAA fairly consistently but at a lesser magnitude than the mice on 60% CR. In this group, FAA was statistically significant on CR days 14 and 28 at p<0.001, on days 7, 21 and 42 at p<0.05, but not on day 35, although some individual mice did exhibit preprandial high activity behaviors on that day. The mean fraction of high activity that was observed during the FAA window in both AL mice and the mice fed 110% of baseline was consistently below 5% for all days. There was no statistically significant difference between the AL controls and the mice fed 110% at any time point except day 21 when AL mice showed an increase in activity (p<0.05). Waveforms of normalized activity are shown for the first day (Day 0) and the 28^th^ day of modified diet (Figure 3D–E). We also tested for a correlation between percentage of weight loss and FAA within treatement groups but found no evidence for linear relationships as r^2^ values were very low; for example, the r^2^ value from a linear regression between % weight loss and FAA among mice on 60% CR was only 0.09 (Figure 3F). Similar analyses was performed for each day of the experiment with a similar result (Supplemental Figure 1).

### Neural Correlates of Acute and Scheduled Hunger

To determine the patterns of neural activity in mice on CR feeding schedules, additional C57BL/6J male mice were obtained and assigned to one of three groups: 1) 60% CR feeding at ZT 10 for 28 days 2) AL controls given an extra food pellet at ZT 10 daily to control for disturbances and 3) fasted controls treated identically to group 2 until the last day of the experiment, day 29, when food was removed 24 h prior to euthanasia. The acutely fasted mice control for c-Fos activation in response to hunger and any nonspecific wakefulness or activity. Based on our observations in the present study (Figure 3) and those of our prior studies [18], [28], [39] with C57BL/6J male mice, FAA begins ∼3 hours prior to scheduled meal time. On the final day of the experiment, day 29, brain samples were taken from n = 2–4 mice from each group at ZT 2.5 (7.5 hours prior to meal time, indicated as TP −7.5 in the Figures), ZT 5.5 (TP −4.5), ZT 7.5 (TP −2.5), ZT 9 (TP −1), and ZT 11 (2 hours after meal time, labeled as TP +2). The mice at +2 were fed their meal so signals at this time point are either due to entrainment or feeding itself. Brains were fixed, cut into 35 micron sections, and every 6^th^ section was immunostained for c-Fos (see Materials and Methods).

**Figure 4:**
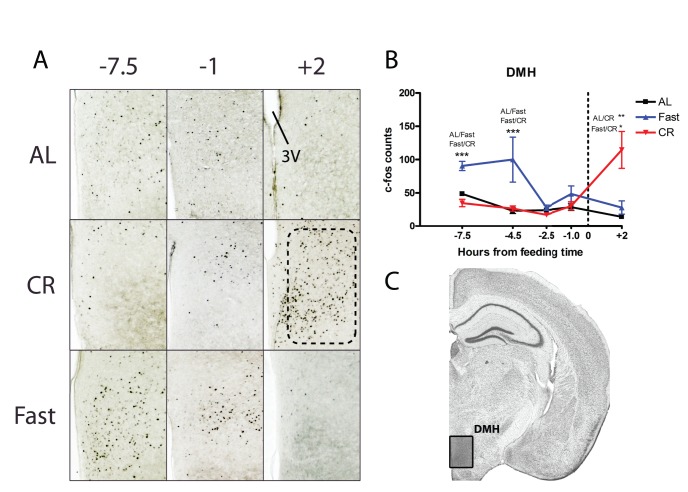
c-Fos protein expression in the dorsomedial hypothalamus of calorie-restricted, acutely fasted, and AL control mice. (A) Representative images of c-Fos staining the DMH of mice at −7.5, −1 hours before feeding and +2 hours after feeding. (B) Mean (±SEM) number of c-Fos-immunoreactive nuclei in the DMH of AL controls (blue), fasted (blue), and CR (red) at 5 time points relative to feeding. TP 0 represents time of expected meal presentation as highlighted by the vertical dotted line on the x-axis. (C) Representative coronal brain map with Nissl stain showing location of region under study. (Adapted from Paxinos and Franklin 2007) [57]. Sample sizes for TP−7.5 thru +2 are n = 6, 11, 11, 9, 5 for AL groups, n = 6, 11, 8, 9, 5 for CR groups, and n = 4, 4, 4, 4, 4 for Fasted groups.

Subsequently, these sections were examined microscopically, focusing on patterns of c-Fos induction in regions of the hypothalamus that have been implicated in metabolism, circadian rhythm, and/or food entrainment. We began by examining the dorsomedial hypothalamus (DMH), which has been reported to express c-Fos and the circadian clock proteins Per1 and Per2 in association with restricted feeding schedules in which food access is limited to 4–6 h in the mid-light period [26], [31] [40], [41]. Counting both the pars compacta and the ventral DMH revealed essentially no c-Fos induction prior to scheduled meal time in mice on CR diets (Figure 4). Most of the c-Fos induction in the fasting group was seen at time point (TP) −7.5 (7.5 hours prior to scheduled meal time) and −4.5 hours where c-Fos was significantly higher in the DMH of fasted mice versus those on Al or on CR (p<0.001). The most striking group difference in c-Fos activation occurred 2 hours after meal time when there were at least twice as many c-Fos positive nuclei in the CR samples compared to the AL or fasted groups (Figure 4; p<0.05 for CR versus fasted; p<0.01 for CR versus AL).

**Figure 5:**
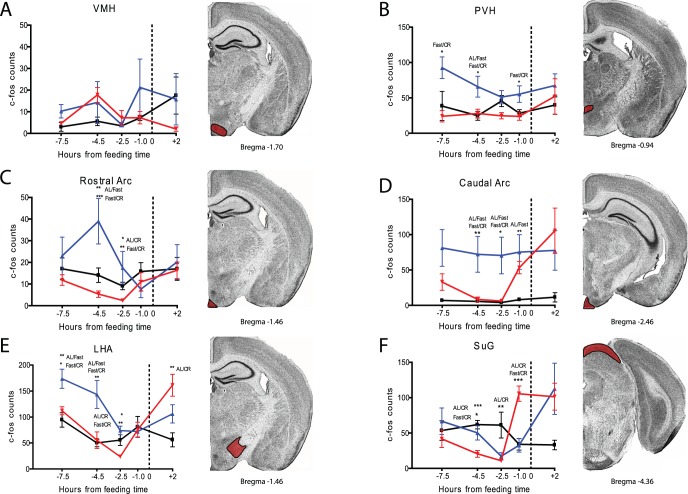
c-Fos induction in VMH, PVH, LHA, Rostral and Caudal Arc, and SuG of calorie-restricted, acutely fasted, and AL control mice. Mean (±SEM) number of c-Fos-immunoreactive nuclei in the several brain nuclei of AL controls (blue), fasted (blue), and CR (red) at 5 time points relative to feeding. TP 0 represents time of expected meal presentation as highlighted by the vertical dotted line on the x-axis. (A) Mean (+/− SEM) c-fos counts for the ventral medial hypothalamus (VMH). (B) c-Fos counts for the paraventricular nucleus (PVH). (C) c-fos counts for the rostral arcuate nucleus. (D) c-fos counts for the caudal arcuate nucleus. (E) c-fos counts for the lateral hypothalamus (LHA). (F) c-fos counts for the superior colliculus gray layer (SuG). Sample sizes for TP−7.5 thru +2 are n = 2–6, 7–12, 7–12, 6–9, 4–6 for AL groups, n = 3–6, 4–12, 5–12, 4–9 for CR groups, and n = 5–6, 5–6, 2–6, 6, 5–6 for Fast groups. Representative images of each neuroanatomical location are shown (from Paxinos and Franklin).

We continued by examining other hypothalamic structures that have been implicated in FAA, hunger sensing, or both. The ventromedial hypothalamus (VMH) was implicated as a mediator of FAA by Ribeiro and colleagues using mice [32] but our cell counts in this region showed no significant group differences at any time point (Figure 5A). We next examined the paraventricular nucleus of the hypothalamus (PVH), a structure that showed c-Fos activation prior to a scheduled daytime meal in rats [41]. Our results clearly indicate an increase in c-Fos expression in this region in fasted mice but no change in the mice on CR feeding (Figure 5B). We examined the arcuate nucleus (Arc) separately in terms of rostral and caudal axes [42]. AL and CR samples showed similar c-Fos expression before mealtime in the rostral Arc, but fasted mice showed a significant increase at −4.5 and −2.5 hours before feeding (Figure 5C; p<0.05 AL vs. CR and p<0.01 Fast vs. CR). In the caudal Arc we observed increased c-Fos expression in the CR group one hour prior to mealtime relative to earlier time points, but not relative to the AL controls (Figure 5D). Interestingly, the caudal Arc showed strong and continued activation in fasted mice at all time points, including after feeding at +2 hours. The lateral hypothalamus (LHA) showed an activation pattern similar to that of the PVH with fasted samples significantly elevated over AL and CR at −7.5 and −4.5 hours prior to feeding (Figure 5E). A notable difference occurred after feeding, when CR samples showed a large increase in c-Fos expression compared to AL samples in the LHA, similar to what was observed in the DMH (Figure 5E). During our initial survey we noted that the gray layer of the superior colliculus (SuG) showed strong c-Fos induction one hour prior to feeding (Figure 5F). The CR group showed a strong and statistically significant c-Fos induction in the SuG one hour prior to meal time when values surged from about 10 cells per section to more than 100 (p<0.001 at TP −1 comparing CR to AL or to fast; Figure 5F). In contrast, the fasted mice showed an increase in staining in the SuG only after feeding. Given its role in visual processing, this region may be a correlate of arousal rather than a mediator of FAA.

**Figure 6:**
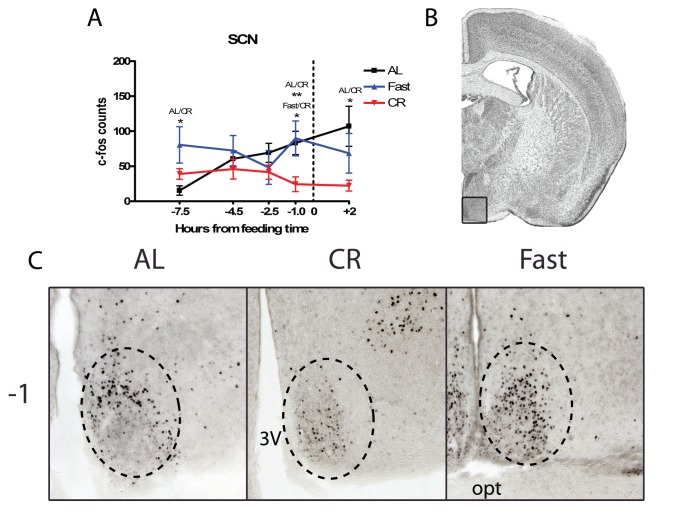
c-Fos protein expression in the in the suprachiasmatic nucleus (SCN) of calorie-restricted, acutely fasted, and AL control mice. (A) Mean (±SEM) number of c-Fos-immunoreactive nuclei in the SCN of AL controls, fasted, and CR at 5 time points measured relative to feeding. (B) Representative coronal brain map with Nissl stain showing location of region under study adapted from Paxinos and Franklin. (C) Representative micrographs at TP−1 of FOS expression in the SCN. Sample sizes for TP−7.5 thru +2 are n = 6, 11, 12, 8, 6 for AL groups, n = 5, 12, 11, 11, 6 for CR groups, and n = 6, 6, 6, 6, 6 for Fast groups.

The last hypothalamic structure that we examined was the SCN, which despite being ruled out as a circadian clock necessary for FAA, may be a modulator of FAA and is thought to compete with the FEO for control over the arousal state of the animal. CR treatment groups show a significantly lower preprandial c-Fos induction at TP −1 compared to both fasted and AL mice (p<0.05 and p<0.01 respectively) (Figure 6). Suppressed c-Fos induction in the CR mice continued into the post-prandial time point, with AL mice showing significantly higher c-Fos induction compared to the CR group (p<0.05), and the fasted group showing a trend for more c-Fos cells compared to the CR group.

**Figure 7:**
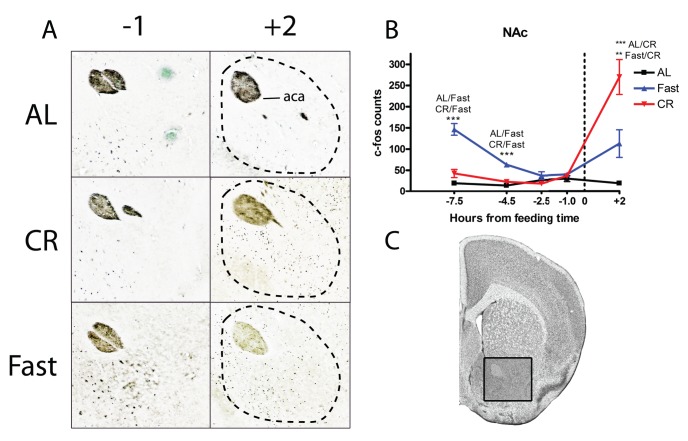
c-Fos protein expression in the in the nucleus accumbens core (NAc Core) of calorie-restricted, acutely fasted, and AL control mice. (A) Representative micrographs of c-Fos staining in the core of the nucleus accumenbs at TP−1 and +2. (B) Mean (±SEM) number of c-Fos-immunoreactive nuclei in the NAc Core of AL, fasted, and CR mice at 5 time points measured relative to feeding. (C) Representative coronal brain map with Nissl stain showing location of region under study. (Adapted from Paxinos and Franklin). Sample sizes for TP−7.5 thru +2 are n = 4, 8, 8, 6, 4 for AL groups, n = 4, 8, 8, 6, 4 for CR groups, and n = 4, 4, 4, 4, 4 for Fast groups.

We continued our search for neural correlates of food entrainment outside of the hypothalamus by examining the nucleus accumbens core (NAc), an area involved in reward and the motivational drive for caloric intake. One prior study in rats implicates the NAc in food entrainment [43]. Our c-Fos counting results in the NAc were similar to those obtained in the DHM and LHA. There was no preprandial c-Fos induction in mice on CR (Figure 7). In contrast, fasted mice showed a significant increase in c-Fos expression at −7.5 and −4.5 hours before feeding (p<0.001 fast versus AL or CR). The NAc showed a massive increase in c-Fos staining after feeding in CR mice and in fasted mice as well but not as large of an increase (p<0.001 CR versus AL, p<0.01 CR versus fasted; Figure 7).

**Figure 8:**
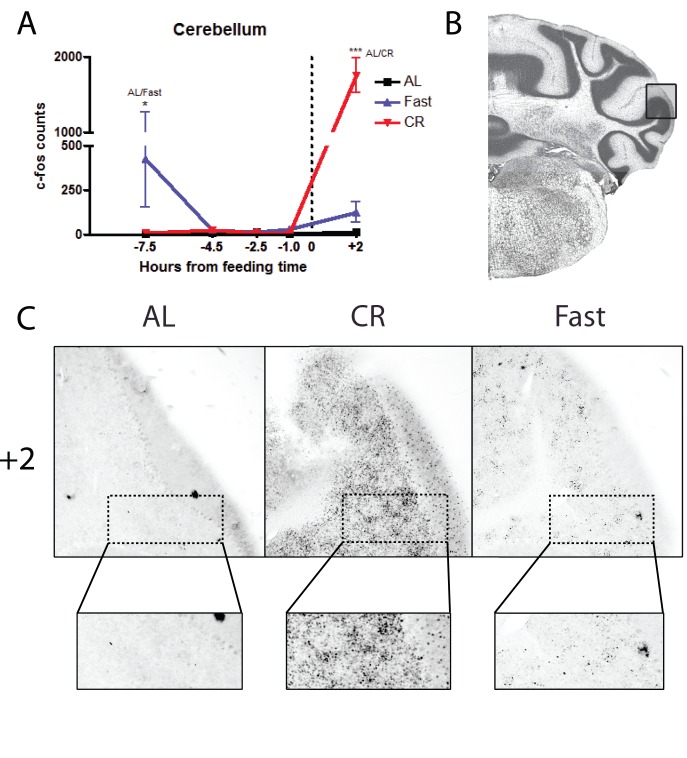
c-Fos protein expression in the in the ansiform lobule crus 2 of the cerebellum of calorie-restricted mice, acutely fasted, and AL control mice. (A) Mean (±SEM) number of c-Fos-immunoreactive nuclei in the NAc Core of AL controls, fasted and CR mice at 5 time points measured relative to feeding. CR mice show a prodigious increase (p<0.001) in FOS protein expression at TP+2 (note the split y-axis). Fasted show significant induction of FOS protein at TP−7.5 (p<0.05). AL control mice do not show any appreciable induction in the expression of FOS protein at any time point. (B) Representative coronal brain map with Nissl stain showing location of the ansiform lobule crus 2 of the Cerebellum adapted from Paxinos and Franklin. (C) Representative micrographs at TP+2 of FOS expression. Insets show high magnifications of c-Fos labeling in the lobule. Sample sizes for TP−7.5 thru +2 are n = 6, 15, 10, 8, 3 for AL groups, n = 6, 12, 12, 9, 6 for CR groups, and n = 3, 5, 5, 5, 3 for Fast groups.

The final structure that we examined was the cerebellum, which may contain FEOs [44]. We quantified c-Fos expression in the ansiform lobule crus 2 of the cerebellum. We observed no preprandial increase in c-Fos staining in CR samples (Figure 8). However, after feeding there was a massive increase in c-Fos immunostaining in the cerebellum (Figure 8) (p<0.001 CR versus AL, at TP +2). Although we quantified only the ansiform lobule crus 2, visual inspection of other areas of the cerebellum showed similar changes.

## Discussion

In this study we observed that acute fasting specifically increases nocturnal activity. We have determined that timed 60 and 80% CR feeding induces robust FAA in the hours before feeding and that this increased activity before meal time comes at the expense of nocturnal activity. The results of our dose response study emphasize the important role of caloric intake in eliciting FAA. Although mice on a relatively modest CR such as 80% CR will exhibit FAA, this difference becomes small after 14 days of scheduled feeding. In addition, suppression of nocturnal activity of mice on 80% CR ceases after only 14 days of CR. This shows that mice on a modest CR not only exhibit reduced FAA relative to mice on a more severely restricted diet but also exhibit daily activity patterns that may gradually become more like those of mice on an AL diet over the course of an experiment. Our results suggest that a 60% CR is ideal for maintaining consistency in behavior across individual mice as well as over the course of experiments lasting several weeks. Whether the increased FAA in 60% CR mice relative to 80% CR is due to effects downstream from FEOs driving FAA or also involves an increase in the strength of food as a Zeitgeber is an open question that would require range of entrainment or meal-shift re-entrainment experiments to assess.

Using the 60% CR protocol we then examined neural correlates of daily feeding or acute fasting using c-Fos immunostaining. Surprisingly, we observed very little evidence for preprandial activation of brain regions in mice on CR schedules, but did observe dramatic activations of many brains regions in CR groups when samples were taken two hours after meal onset. These results are striking in the magnitude of the differences before and after mealtime, and in the discrepancies with previous studies that have reported c-Fos activation of a number of these structures in rats or mice on limited-time as opposed to limited-amount feeding schedules. One factor that could be explored in future studies is the number of days of restricted feeding prior to c-Fos assessment. Mice in our study were adapted to restricted feeding schedules for 1 month, while previous studies have typically used 10, 14 or 21 days. c-Fos in some brain regions may be activated by metabolic factors that eventually adapt to long-term caloric restriction. This may also explain the increased c-Fos expression noted in the acute 24 h fasting groups in most brain regions sampled in our study.

The DMH has been the focus of numerous studies of FAA employing lesion and clock gene or immediate early gene mapping methods [40], [45], [46], [47], [48]. At least three studies using limited-duration daytime feeding schedules reported significant increases of c-Fos prior to mealtime in rats or mice [41]. Our data, by contrast, reveal no preprandial induction in the DMH of mice maintained on a 60% CR schedule but strong postprandial c-Fos induction (consistent with postprandial data from Poulin and Timofeeva, 2008) and strong induction in fasted groups at the very early preprandial time points, suggesting a predominantly satiety-sensing or meal processing role for the DMH in our model. While the DMH has been ruled out as necessary for FAA, it has been proposed as a source of inhibitory inputs to the SCN pacemaker, which promote the expression of FAA by suppressing sleep-promoting SCN output during the day (the sleep phase in nocturnal rodents) [40], [47], [49]. Our immunohistochemical data do not provide strong support for a DMH-SCN intrahypothalamic gate model given the visibly low c-Fos induction in the DMH in both CR and AL groups at all preprandial time points.

On the other hand, we did observe significantly lower c-Fos induction in the SCN prior to mealtime in the CR groups compared to Fast/AL groups. This result is consistent with the model that FAA may be facilitated by inhibition of the SCN to permit locomotor activity in the usual sleep phase of the circadian cycle. This model is further supported by evidence that FAA is enhanced by SCN-ablation in rats [50]. We previously observed that AL fed mice can anticipate a daily palatable meal of cheese or high fat diet and that this was associated with a modest increase in c-Fos expression in the SCN [28] This suggests that mice can anticipate a daily meal without SCN inhibition (or, more precisely, without a reduction of c-Fos protein in the SCN). A difference in pre-meal SCN activity between CR and palatable feeding schedules may explain in whole or in part why FAA is much greater in mice on the CR schedules.

The nucleus accumbens, part of the reward system, has been another target of interest regarding a role in promoting FAA. Mendoza and colleagues [43] observed c-Fos induction in the NAc prior to mealtime, and found that lesions restricted to the NAc suppressed FAA in rats. An earlier study in which the entire NAc was removed did not observe reduced FAA in food restricted rats, indicating that while components of the NAc may participate in expression of FAA, the structure cannot be the source of signals critical for timing FAA [51]. A modulatory role for the NAc is also suggested by the results of recent studies of ghrelin-deficient mice, which expressed less FAA in some studies, and have reduced c-Fos expression in the mesolimbic dopamine pathway under restricted feeding conditions [29]. Our c-Fos data are not consistent with a critical role for the NAc given the extremely low preprandial c-Fos induction in mice on CR. On the contrary, the strong induction of c-Fos 2 hours after meal onset is more consistent with a role for the NAc in processing stimuli related to food intake, including its reward properties. The NAc may be more important for anticipating palatable food [30], [52], although this remains to be tested directly.

In conclusion, the results of our study underscore the importance of feeding schedule parameters for the expression of behavioral and neural correlates of FAA in mice. Mice provided food at the same time every day may show weak FAA if CR is modest. This raises the possibility that neural, genetic or endocrine manipulations could reduce FAA without impinging on the food-entrainable clock mechanism, by reducing metabolic activity and thereby effectively reducing the degree of CR expressed relative to caloric needs. The results of our study also raise questions about the role of several hypothalamic structures thought to be important for induction of FAA. We established that a 60% CR schedule induces robust FAA in mice, but using this schedule failed to observe food anticipatory increases in c-Fos in the DMH, VMH, PVN, Arc, or LHA of mice after ∼30 days of restricted feeding. By contrast, most of these structures showed robust elevation of c-Fos 2-h after meal onset. These results demonstrate that neural activity in the hypothalamus and other regions can be dissociated from FAA in CR mice, as has previously been reported in rats and mice anticipating a palatable daily snack without CR [28], [30], [53]. In the absence of convincing evidence that FAA is eliminated by lesions confined to any one brain structure, many researchers in the field now favor the idea that FAA is regulated by a distributed population of FEOs [2], [9], [30], [33], [54], [55], [56]. This model would seem to predict activation of multiple regions in the brain preceding scheduled meal time, but this was not evident in our study. It is certainly possible that FAA output may involve reductions in activity in some structures (e.g., the SCN) and activation in other structures that is not reflected in marked c-Fos expression, or there may have been c-Fos entrainment in structures not thoroughly examined in this study (e.g., brain stem nuclei). This issue may require alternative measures of neural activity for clarification.

## Supporting Information

Figure S1 **Correlation of normalized food anticipatory activity in the 3 hours preceding scheduled feeding with % body weight loss at Day 7 (A), Day 14 (B), Day 21 (C), Day 35 (D), and Day 42 (E).**
(EPS)
